# Supplementary material for: Content-rich biological network constructed by mining PubMed abstracts
Source: BMC Bioinformatics. 2004 Oct 8;5:147. doi: 10.1186/1471-2105-5-147 (PMC528731; doi:10.1186/1471-2105-5-147)
Supplement: Additional File 2 — The original results of the above study (non-essential files are deleted to keep the file size under the limit set by BMC bioinformatics). [file 1471-2105-5-147-S2.bz2 › chilibotAdditionalFile2/dip05/333ID8171323E3969/html/TNF_LTBR.html]

 


 **TNF** and **LTBR** 
  
Found 627 abstracts in PubMed, retrieved 05.  
 

 What does Google say? 
 PDF only 
| .edu only 

---

**Interactive relationship** (e.g. stimulation, inhibition, etc)

**Neutral relationship**- LT consists of soluble LT alpha 3, which binds to  **TNF**  R1 and  **TNF**  R2, and membrane LT alpha 1 beta 2, which binds to LT beta R  [ **LTBR** ] .  Ref: 12882833 Int Immunol, 2003

**Non-interactive relationship** (e.g. studied together, co-existance, homology, etc.)

- After treatment, the UAER, CD11b CD18  [ **LTBR** ]  expression and  **TNF**  alpha  [ **TNF** ]  concentration lowered significantly in the treated group P <.  Ref: 12872395 Zhongguo Zhong Xi Yi Jie He Za Zhi, 2003
- The changes of urinary albumin excretion rate UAER, CD11b CD18  [ **LTBR** ]  expression and tumor necrosis factor alpha  [ **TNF** ]   **TNF**  alpha  [ **TNF** ]  concentration before and after treatment were observed.  Ref: 12872395 Zhongguo Zhong Xi Yi Jie He Za Zhi, 2003
- Gene knockout study suggests that NIK is dispensable for  **TNF**  R1 but required for lymphotoxin beta receptor  [ **LTBR** ]  induced NF kappaB activation.  Ref: 12853971 Oncogene, 2003
- This activity of LT depends on membrane LT alpha 1 beta 2 and not soluble LT alpha 3, since DA virus persisted and induced inflammatory demyelination in LT beta R  [ **LTBR** ] , but not  **TNF**  R1 or  **TNF**  R2, mice.  Ref: 12882833 Int Immunol, 2003
- RESULTS The CD11b CD18  [ **LTBR** ]  expression and  **TNF**  alpha  [ **TNF** ]  concentration in DM patients were higher than those of normal range P <.  Ref: 12872395 Zhongguo Zhong Xi Yi Jie He Za Zhi, 2003
- Glands that received AdTNFRp55 Ig concurrently with activated PBLs had decreased numbers of CD4 cells, CD18  [ **LTBR** ]  cells, RTLA, and apoptotic cells.CONCLUSIONSIn vivo transduction of the lacrimal gland with AdTNFRIp55 Ig resulted in transient expression in the gland and the appearance of  **TNF**  inhibitor protein in tears.  Ref: 12792478 Cornea, 2003
